# Environmental Pollutant Benzo[a]pyrene Induces Recurrent Pregnancy Loss through Promoting Apoptosis and Suppressing Migration of Extravillous Trophoblast

**DOI:** 10.1155/2020/8983494

**Published:** 2020-10-16

**Authors:** Yang Ye, Sushi Jiang, Chao Zhang, Yanxiang Cheng, Huan Zhong, Tao Du, Wenming Xu, Ricardo Azziz, Huidong Zhang, Xiaomiao Zhao

**Affiliations:** ^1^Department of Obstetrics and Gynecology, Sun Yat-sen Memorial Hospital of Sun Yat-sen University, Guangzhou, 510120 Guangdong, China; ^2^Guangdong Provincial Key Laboratory of Malignant Tumor Epigenetics and Gene Regulation, Sun Yat-sen Memorial Hospital of Sun Yat-sen University, Guangzhou, 510120 Guangdong, China; ^3^Department of Pharmacology, Cardiac & Cerebral Vascular Research Center, Zhongshan School of Medicine, Sun Yat-sen University, Guangdong, China; ^4^Department of Obstetrics and Gynecology, Renmin Hospital of Wuhan University, 99 Zhang Zhidong Road, Wuhan 430060, Hubei, China; ^5^State Key Laboratory of Pollution Control and Resources Reuse, School of the Environment, Nanjing University, Nanjing 210023, China; ^6^Environmental and Life Sciences Program (EnLS), Trent University, Peterborough, Ontario, Canada; ^7^Key Laboratory of Birth Defects and Related Diseases of Women and Children (Sichuan University), Ministry of Education, Sichuan University, Chengdu610041, China; ^8^Academic Health and Hospital Affairs, State University of New York (SUNY) System Administration, New York 12246, USA; ^9^Key Laboratory of Environment and Female Reproductive Health, West China School of Public Health & West China Fourth Hospital, Sichuan University, Chengdu 610041, China

## Abstract

**Methods:**

The implantation sites, fetus resorption, and abnormal fetuses were studied in pregnant mice treated with different doses of BaP by oral gavage from day 1 to day 10 of gestation. Additionally, apoptosis and related signaling pathway, and the migration and invasion of trophoblasts, were assessed before and after exposure of BPDE in Swan 71 trophoblast cell. Besides, the migration and invasion, and its related signaling pathway, were assessed in villi obtained from women.

**Results:**

We observed a concentration-dependent incidence of abnormal murine fetuses, beginning with 0.1 mg/kg BaP; with a BaP concentration of 2 mg/kg, no fetuses developed. Correspondingly, a BPDE concentration-dependent apoptosis of human trophoblasts. Beginning with 0.5 *μ*M BPDE exposure, Bax/Caspase-3 were increased and Bcl-2 decreased. Furthermore, BPDE also inhibited, in a dose-dependent manner, the migration of villous explants from elective abortion women, consistent with the reduced migration of villous explants from women with recurrent pregnancy loss (RPL), and reduced the cell immigration in Swan 71 trophoblasts, in a dose-dependent manner measured by transwell assays.

**Conclusions:**

Our study results provide mechanistic insight to the effect of BPDE on trophoblast dysfunction through enhanced cell apoptosis and inhibited migration, providing further experimental evidence to the causative links between BaP exposure and PRL.

## 1. Introduction

There is a growing concern about the effects of environment endocrine-disrupting chemical (EDC) exposure on the human reproduction. Studies have found that some negative pregnancy outcomes may be related to representative polycyclic aromatic hydrocarbons (PAH) exposure [1]. Benzo(a)pyrene (BaP), the most representative PAH, is present at relatively high levels in cigarette smoke, petroleum products, charbroiled foods, and contaminated water [2]. BaP can be absorbed via the oral, inhalation, and dermal routes of exposure [3]. After absorption, BaP is activated by the aromatic hydrocarbon receptor-induced cytochrome P4501A1 and epoxide hydrolase and metabolized as carcinogenic benzo[a]pyren-7,8-dihydrodiol-9,10-epoxide (BPDE) [4]. Epidemiological studies have found that exposure to elevated levels of PAHs may be associated with lower serum levels of progesterone, reduced fetal growth [5], and higher risk of early miscarriage [6], implying it may interfere in the reproductive process [7]. The population analysis also suggested that BaP exposure was associated with the reduced fetal birth weight and length, head circumference, preterm birth, congenital anomalies, and growth restriction [3, 8, 9].

In addition, animal studies suggested that different doses of BaP exposure may have different toxicities. For example, relative low concentration of 0.2 mg/kg/d BaP exposure to female mice inhibited apoptosis of endometrial stromal cells during early pregnancy, resulting in uneven sized implantation sites. When the BaP exposure increased to 2 mg/kg/d, all the implantation sites were totally lost [10].

Recurrent pregnancy loss (RPL), defined as loss of consecutive two or more than two pregnancies before 20 weeks of gestation, occurs in 1-5% women of reproductive age [11]. Trophoblasts play a critical role in the implantation and formation of the maternal-fetal interface; thus, their function is essential for maintaining of normal pregnancy [12]. During embryo implantation, the maternal-fetal interface is in highly dynamic balance through the close mediation of proliferation and apoptosis of trophoblasts [13]. If out-of-balance apoptosis in trophoblasts happens in pathological conditions, placental development may be impaired, leading to adverse pregnancy outcomes such as pre-eclampsia and miscarriages [14]. Furthermore, in the process of implantation, trophoblast cells penetrate through the endometrial epithelium and intrude into the stroma layer, followed by the differentiation and spiral artery remodeling [15]. Thus, the impaired trophoblast migration and invasion often lead to feeble maternal-fetal linkage establishment and are connected with diseases such as RPL, pre-eclampsia, and preterm labor [16]. However, the specific effect of BaP and its metabolite BPDE on trophoblast, and subsequently, on pregnancy loss, remains unknown. In this study, we hypothesize that it may induce the apoptosis or inhibit the migration of trophoblast, resulting in the occurrence of miscarriage.

In this study, concentration-dependent occurrence of reduced implantation sites, more fetus resorption, and abnormal fetuses were observed in BaP-treated pregnant mice. Subsequent cell experiments demonstrated that BPDE activated Bax/Caspase while it reduced Bcl-2 expression in Swan 71 trophoblast cell. In addition, BPDE inhibited the invasion of villous explants, consistent with the reduced migration of villous explants in RPL women. BPDE reduced Swan 71 migration in a dose-dependent manner, probably through the PI3K/AKT/MMP2 signaling pathway. Our research may provide a new direction to illustrate the reproductive toxicity of BaP and BPDE.

## 2. Materials and Methods

### 2.1. Materials

Benzo[a]pyren-7,8-dihydrodiol-9,10-epoxide (BPDE) was purchased from MRIGlobal Chemical Co. (Kansas, Missouri, USA). BPDE was dissolved in DMSO as 4 mM stock and stored at in single-use aliquots. Before the experiment, BPDE stock fluid was dissolved into each concentration freshly using 10% FBS, DMEM/F12. All vehicle control and treated cultures contained the identical amount of DMSO (0.01%, *v*/*v*). BaP (purity, 96%) and its solvent corn oil were purchased from Sigma-Aldrich (St. Louis, MO). All chemicals were of analytical grade.

### 2.2. Animals and BaP Administration

The study protocols were approved by the Ethics Committee of Zhongshan School of Medicine, Sun Yat-sen University on Laboratory Animal Care. 9 to 12-week-old C57BL/6 mice were purchased from the Laboratory Animal Center of Sun Yat-sen University. After 7 days of adaptation, virgin female mice were mated with fertile males (3 : 1) overnight and examined for the presence of the vaginal plug as the sign of pregnancy the following morning; the day upon which a vaginal plug was observed was designated “D1 of gestation.” Pregnant mice were administered daily with corn oil (control) or different concentrations of BaP (0.05, 0.1, 0.2, and 2 mg/kg, dissolved in corn oil) by oral gavage at 0.1 ml/10 g of body weight from D1 to the day of sacrifice. The mice were decapitated between 08:00 and 09:00 on D8 or D10, and the implantation sites, pregnant sacs, and uteri were collected after morphological observations. Morphological observations were performed independently by two persons who were blinded to the group assignment of the mice.

1. Abortion: no pregnant sacs in pregnant mice, defined as emergence of vaginal plugs
2. Resorption fetuses: implantation sites containing no intact fetus inside the placenta
3. Abnormal fetuses: fetuses which appeared intact but were considerably smaller than their normal littermates or normal fetuses from other mice
4. Resorptions: implantation sites containing no intact fetus inside the placenta
5. Abortion rate: number of pregnant mice without implantation site/number of pregnant mice with vaginal plugs × 100%
6. Percentage of abnormal fetuses: number of abnormal fetuses/total number of fetuses observed × 100% [17]

### 2.3. Cell Culture and Treatment

Human first trimester human extravillous trophoblast cell line Swan 71 was cultured in Dulbecco's modified Eagle's medium/F12 supplemented with 10% (*v*/*v*) fetal bovine serum plus Penicillin/Streptomycin antibiotics (Biological Industries, Israel) and incubated in a humidified atmosphere at 37°C with 5% CO_2_. Swan 71 cells were seeded in 6-well culture plates, then incubated overnight, and exposed to 0.25, 0.5, 0.75, and 1.0 M BPDE for 24 h.

### 2.4. RNA Preparation and Quantitative Real-Time PCR

Total RNA was extracted Swan 71 cell by an RNAiso Plus kit (TaKaRa, Japan) according to the manufacturer's instrument and measured by NanoDrop One (Thermo Scientific, USA). RNA was reverse-transcribed by a PrimeScript™ RT Master Mix kit (TaKaRa, Japan). Quantitative real-time PCR analysis (Light Cycler 480, Roche, Germany) was performed to detect the expression of RNA using a TB Green kit (TaKaRa, Japan). The related PCR primers are presented in Table 1.

### 2.5. Western Blot Analysis

Proteins from cells were extracted using radioimmunoprecipitation assay (RIPA) lysis buffer (CWBIO, China) with protease and phosphatase inhibitors (CWBIO, China). Identical quantities of proteins were electrophoresed by SDS-PAGE, transferred onto PVDF membranes, blocked with 5% non-fat milk for 1 h, and incubated with primary antibodies specific for Bax, Bcl-2, Caspase-3 (#2772, #2872, and #9662) (1 : 1000, Cell Signaling Technology, USA), and *β*-actin (66009-1-Ig, 1 : 5000, Proteintech, USA) at 4°C overnight, followed by incubation with anti-rabbit/mouse HRP-conjugated secondary antibodies (cw0103s, cw0102s) (1 : 2000, CWBIO, China) at room temperature for 1 h. Signals were detected by Immobilon ECL substrate (Millipore, Germany).

### 2.6. Cell Migration Assay

Migration assays were carried out using 24-well transwell inserts equipped with 8 *μ*m pore size membrane (Corning). Swan 71 trophoblast cells were exposed to different doses of BPDE for 24 h. The floating dead cells were removed by washing, and only the survived cells were used for the following assays. The survived Swan71 trophoblast cells were seeded into the upper chamber, and cell migration was detected. DMEM/F12 media containing 10% FBS was used as a chemoattractant in the lower chamber. After 24 h, the upper chambers were removed, and the cells on the upper side of the membrane were completely removed through gentle swabbing. The remaining cells that migrated were fixed with 4% paraformaldehyde for 15 min and stained with 0.5% crystal violet. The migrated cells in each well were photographed with a microscope at 100x magnification and counted by ImageJ. All experiments were replicated thrice.

### 2.7. Flow Cytometry

Apoptotic cells were detected by Annexin V-fluorescein isothiocyanate (FITC)/propidium (PI) double staining using Annexin V-FITC Apoptosis Detection Kit (Keygene Biotech, Nanjing, China) according to the manufacturer's instruction. Briefly, cells were collected with cold phosphate-buffered saline (PBS) and then stained with Annexin V-FITC and PI in binding buffer for 20 minutes. The stained cells were then analyzed using CytoFLEX (Beckman Coulter, California, USA).

### 2.8. Villous Explant Culture

The study protocol was approved by the Ethical Committee of Sun Yat-sen Memorial Hospital, Sun Yat-sen University. All patients completed an informed consent form to collect tissue samples. The explant culture was performed as described previously [18]. In brief, tips of first trimester placental villi (6-10 weeks) were collected from women with elective abortion health control (HC) or RPL groups and stored in 4°C sterilized normal saline. Within 0.5 h after collection, villi were dissected into small 2 or 3 mm tissue sections and explanted in 24-well culture dishes precoated with phenol red-free Matrigel substrate (Corning). The dissected tissue pieces were carefully put on top of each drop and incubated to allow anchorage, then were supplemented with 10%FBS and DMEM/F12 medium. Villi successfully anchored on Matrigel matrix and initiated outgrowth and were treated with different concentration of BPDE media or vehicle alone. Images were obtained after 24 h to 72 h of in vitro culture under a light microscope, and the distance between villous tissue and its distal end was measured via ImageJ software to determine the extent of migration. For each villus, 5 explants were cultured and analyzed, and experiments were repeated three times.

### 2.9. Statistical Analysis

Statistical analyses were carried out using GraphPad Prism 7.0. All data were analyzed by Student's *t*-test or analysis of variance. The data are presented as the mean ± standard error of the mean (SEM). *p* < 0.05 was considered statistically significant.

## 3. Results

### 3.1. Mice Exposed to BaP Resulted in Fewer Implantation Sites and Were Likely to Exhibit More Fetus Resorption and Abnormal Fetuses in a Dose-Dependent Manner

**Figure 1:**
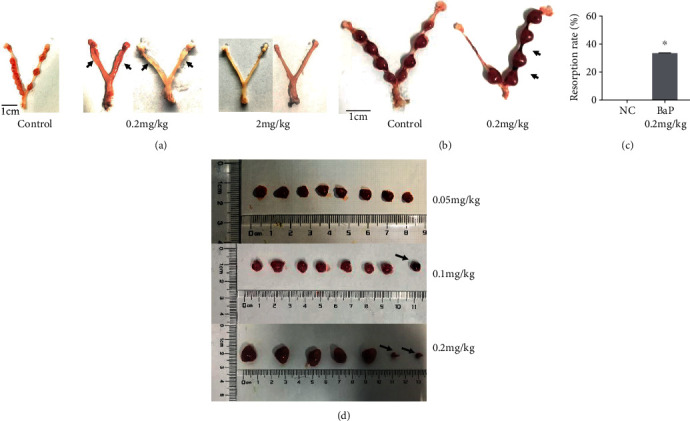
BaP-treated mice exhibited a reduced number of implantation sites and increased abnormal fetuses. (a) The images showed the morphology of mouse uteri in the control and BaP-treated groups on day 8 of gestation. Arrows show no sign of implantation sites, and only swelled uterus could be observed. (b) The image shows the morphology of uteri of control and 0.2 mg/kg BaP-treated mice on day 10 of gestation. Treated mice had five normal fetuses and two resorption sites (arrow). (c) The resorption rates of fetuses in the control and treated groups. ^∗^*p* < 0.05 indicates significant difference from the control group. (d) The image indicates the abnormal fetus numbers increasing with the levels of BaP exposure increasing.

The *in vivo* experiment was used to test the toxic effects of BaP on early pregnancy. We collected the uteri from the mice on day 8 of gestation, and the morphology is presented in Figure1(a). Compared with the control group, which exhibited evenly sized implantation sites, the mice exposed to 0.2 mg/kg BaP exhibited abnormal implantation sites, and those exposed to 2 mg/kg BaP displayed no trace of pregnancy (Table 2). Since days 8-10 of gestation are a critical period for implantation and placentation [17], some mouse exposure to BaP was extended to day 10 of gestation. Mice exposed to 0.2 mg/kg BaP presented 7.5 normal fetuses and 2.5 resorption sites on average, and the resorption rate is 33.3%, while control mice presented no resorption site (Figures 1(b) and 1(c)). The results suggested that BaP might induce fetus resorption and miscarriage by the adverse effect on villi of mice. Furthermore, the percentage of abnormal fetuses were increased as the mice were exposed to BaP increasing from 0.05 mg/kg to 0.2 mg/kg (Table 3, Figure 1(d)). Generally, BaP induced miscarriage and fetus resorption and acted as a teratogen to fetuses in pregnant mice, with its effects intensifying if the BaP exposure was increased.

### 3.2. BPDE Induced Apoptosis in Swan 71 Trophoblast Cell by Activation of Caspase-3 through Bcl-2/Bax Signaling Pathway

**Figure 2:**
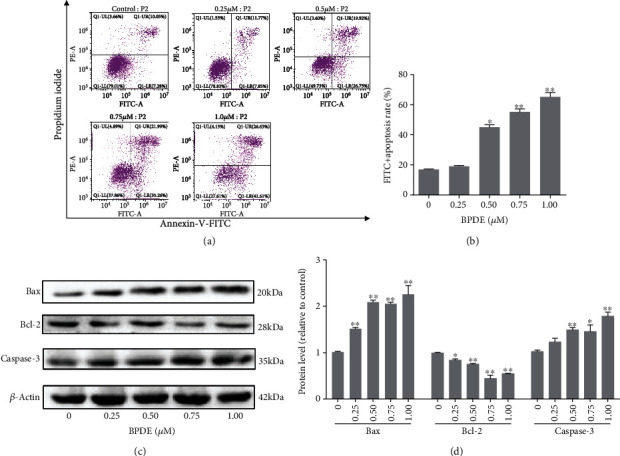
BPDE promotes apoptosis of Swan 71 cell through activating Bax and Caspase-3 while reducing Bcl-2 expressions. (a) Swan71 cells were exposed to 0, 0.25, 0.5, 0.75, or 1.00 *μ*M BPDE for 24 h, and the apoptosis was measured by flow cytometry. (b) The quantitative statistics of FITC+ apoptosis rates. ^∗^*p* < 0.05 or ^∗∗^*p* < 0.01 indicates significant difference from the control cells. (c) The protein levels of Bax and Caspase-3 in Swan 71 cell treated by different concentration of BPDE were measured by Western blotting. (d) The quantitative statistics of scale value, representing relative protein level, determined by ImageJ. ^∗^*p* < 0.05 or ^∗∗^*p* < 0.01 indicates significant difference from the control cells.

Since apoptosis is much more evident in the human villous tissue from patients with RPL, we investigated the effect of BPDE on the apoptosis of trophoblasts using Swan 71 cell line. Swan 71 cells were exposed to 0.25-1.00 *μ*M of BPDE for 24 h, followed by Annexin V and PI dual staining and flow cytometry. By calculating cell percentages in the LR (FITC+PI-) and UR (FITC+PI+) regions, which represented early and later stages of apoptosis, relative apoptosis rates were determined. Cell apoptosis was significantly increased after exposure to BPDE in a dose-dependent manner. (Figures 2(a) and 2(b)). The mechanism underlying the observed effects of BPDE exposure on the apoptosis of Swan 71 cells was investigated using Western blotting. With increased exposure of BPDE, the expression of Bax and Caspase-3 was significantly increased, while Bcl-2 expression was decreased (Figures 2(c) and 2(d)). Collectively, BPDE-induced apoptosis of Swan 71 was regulated by Bax, Caspase-3, and Bcl-2 proteins, and its proapoptotic effects were enhanced as the BPDE exposure increases.

### 3.3. Villous Explants from the RPL Group Exhibited Reduced Migration and Invasion Compared to Those from the HC Group

**Figure 3:**
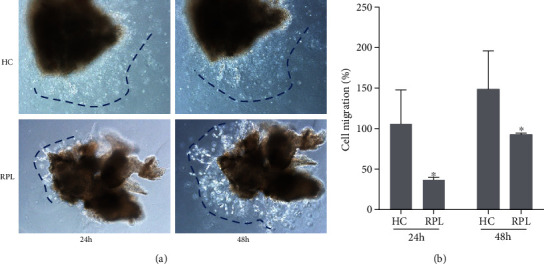
Villous explants from women with RPL show reduced migration and invasion compared to elective abortion health control (HC). (a) Extravillous explants were collected from HC and RPL women, which exclude karyotype, uterine, autoimmune, and endocrine abnormalities, and cultured on phenol red-free Matrigel. At 24 h and 48 h of culture, the distance between villous tissue and its distal end was measured to determine the outgrowth of villous explants. (b) The quantitative statistics of explant migration distance, determined by ImageJ. Migration distance of 24 h cultured villous from HC was determined as 100%. ^∗^*p* < 0.05 indicates significant difference from HC.

Villous explant growth condition can reflect trophoblast migration and invasion [18]. Thus, villous explants from first trimester villi from the RPL and age- and gestational week-matched control groups were cultured on Matrigel-coated dishes. The distance of outgrowth on the Matrigel surface was measured at 24 h and 48 h. Following 24 h of culture, the RPL explants were anchored into the Matrigel and exhibited slower migration compared with the control group at this point. At 48 h of *in vitro* culture, shorter distance outgrowth was observed in RPL explants compared with the control group (Figures 3(a) and 3(b)). The results showed that RPL group explants migrated significantly shorter than the control explants.

### 3.4. BPDE Inhibited Trophoblast Cell Migration through PI3K/AKT/MMP2 Signaling Pathway

**Figure 4:**
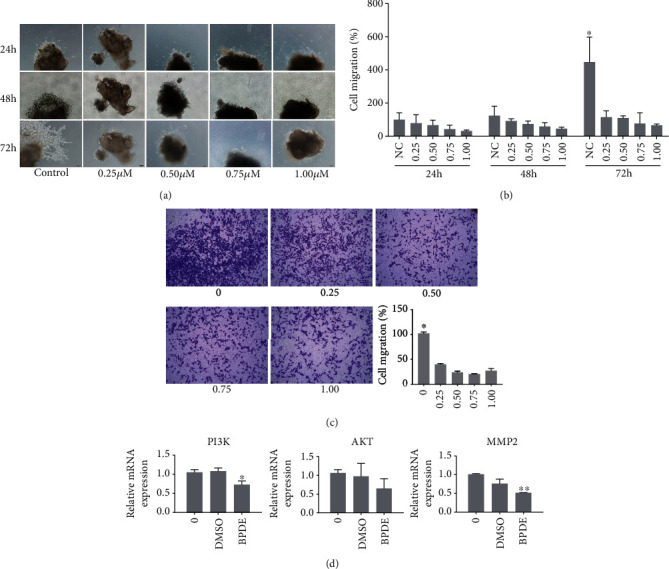
BPDE reduced migration of trophoblast cells in a dose-dependent manner through the PI3K/AKT/MMP2 signaling pathway. (a) The tips of villous tissue by elective abortion patients were exposed to 0, 0.25, 0.50, 0.75, and 1.00 *μ*M BPDE for 24, 48, and 72 h. The BPDE treatment significantly suppressed trophoblast outgrowth from the villous tips compared with control (DMSO) villous explants. (b) The quantitative statistics of explants migration, determined by ImageJ. ^∗^*p* < 0.05 indicates significant difference from the BPDE-treated groups. (c) The image represented migration of Swan 71 cell through the transwell assays after exposure to 0, 0.25, 0.50, 0.75, and 1.00 *μ*M BPDE for 24 h. ^∗^*p* < 0.05 indicates significant difference from the BPDE-treated cells. (d) The mRNA levels of PI3K, AKT, and MMP2 were decreased after exposure to 0.50 *μ*M BPDE for 24 h, determined by qRT-PCR. ^∗^*p* < 0.05 or ^∗∗^*p* < 0.01 indicates significant difference from the control cells.

To confirm the role of BaP metabolic active material benzo[a]pyrene-7,8-diol-9,10-epoxide (BPDE) in trophoblast invasion and migration *in vivo*, we next studied whether BPDE regulates trophoblast outgrowth of villous explants. Tips of HC villi from the same subject were dissected and explanted in 24-wall dishes. 0.25-1.00 *μ*M of BPDE was added to the culture media, and the outgrowth distance of villous explants was monitored at 24, 48, and 72 h. The experiments were repeated thrice. At 24 h of culture, the explants were anchored and no significant difference was observed between the control and BPDE-treated groups (*p* > 0.05). At 48 and 72 h of *in vitro* culture, the explants of the treatment group migrated significantly shorter than the control group explants in a dose-dependent manner (Figures 4(a) and 4(b)). Experiments on the first trimester human extravillous cytotrophoblast-derived Swan 71 cell line further confirmed the results. The Swan 71 cells were exposed to 0.25-1.00 *μ*M of BPDE for 24 h, and the migration ability was determined through transwell assays. Cells in the control group migrated easily, as measured by the number of cells strained by crystal violet. The number of migrated cells decreased in the BPDE-treated group in a dose-dependent manner (Figure 4(c)). Previous studies have suggested that the PI3K/AKT/MMP2 signaling pathway was involved in trophoblast migration [19]. To determine whether BPDE inhibits trophoblast cell migration through this pathway, the mRNA levels of PI3K, AKT, and MMP2 were analyzed by qRT-PCR assays. When compared with the untreated and DMSO-treated control groups, mRNA levels of PI3K and MMP2 in the 0.5 mg/kg BPDE-treated group were significantly decreased (Figure 4(d)). The results demonstrated that the PI3K/AKT/MMP2 pathway may regulate BPDE inhibition of migration of Swan 71 cell.

## 4. Discussion

BaP is regarded as the most abundant environmental pollutant of PAH, widely distributed in polluted air and water as well as in barbecued and fried foods. The human exposure to them is unavoidable. Previous studies in its reproductive toxicity have mainly focused on the ovarian disorders, endometrial cell apoptosis, and decidualization [10, 20, 21]. However, studies on the mechanism of adverse effect on first trimester trophoblasts are scarce. In this study, results demonstrated that mice exposed to BaP resulted in fewer implantation sites, more fetus resorption, and abnormal fetuses in a dose-dependent manner. The mechanism study showed that BPDE might induce apoptosis by activating Bax/Caspase and reducing Bcl-2 in trophoblast cell. Furthermore, BPDE also inhibited the migration of human villous explants and Swan 71 trophoblasts.

Burning solid fuels such as coal lead to a high level of domestic exposure of PAHs, particularly for women who may spend more time cooking [22]. Besides, tobacco smoke and consumption of some plant foods, especially broad-leafed vegetables, are one of the dominant sources of PAH exposure. An epidemiological study has demonstrated that in Chinese city Beijing, the exposure levels to PAHs (*μ*g/person/day) are between 18.3 for male and 15.8 for female adults [23]. In this exposure dose, the accumulated dose of a typical pregnancy woman (60 kg) is 73.7 *μ*g/kg body weight at term delivery day (280 day). In the present study, 0.05-2 mg/kg/day was used to perform the animal BaP exposure experiments, and the accumulation dose is 0.5-20 mg/kg. Using the dose translation equation from animal to human [24], the human equivalent dose is 32-129.7 *μ*g/kg, which is in the range of the female PAH exposure levels illustrated in the human case study. Similar doses have been applied in other animal studies [10], which proved the doses can impair decidualization and decidual angiogenesis. And the effect of BaP and its metabolite BPDE on the function of trophoblast and miscarriage is not clear.

After conforming the dose-dependent effect of BaP [25] on pregnant mice, we focused on the effect of BPDE on the villous explants and trophoblast cells. Villous explant growth conditions can reflect trophoblast migration and invasion. And Swan 71 cell is telomerase-expressing immortal first trimester trophoblast cell and retains the characteristics of primary trophoblasts in trophoblast studies [26]. Previous studies demonstrated that women exposed to cigarette smoking had a significantly higher BaP level (1.32 ± 0.68 ng/ml) in follicular fluid than their nonsmoking counterparts [27]. Exposed to 1-2.5 *μ*M BaP, trophoblast stem cell accumulation was reported to be decreased, and embryo implantation was impaired [28]. After human exposure, BaP could be metabolically converted to BPDE by placenta, and BPDE-DNA adducts are accumulated in the placenta, or BPDE cross the placenta barrier, producing toxicity to fetal development [29]. 0.25-2.0 *μ*M BPDE exposure impaired trophoblast invasion through inhibition of cell filopodia formation [30] and caused disorder of mitochondrial fission/fusion [31]. In this study, we selected 0.25-1.0 *μ*M BPDE to treat human trophoblast Swan 71 cells.

It has demonstrated that apoptosis is abnormally enhanced in the human conceptus in the first trimester in cases of RPL [32]. Furthermore, apoptosis is also related to pregnancy complications such as preeclampsia and intrauterine growth restriction [32]. The Bcl-2 family acts as a critical check-point in apoptosis upstream of irreversible cellular damage, where the release of apoptogenic factors from mitochondria was controlled by it [33]. The Bcl-2 protein family includes the prosurvival cell effector proteins such as Bcl-2 itself and the proapoptotic guardians Bax (Bcl-2-associated X protein) and BAK (Bcl-2 antagonist/killer). Thus, this family can be regarded as a tripartite apoptotic switch [34]. Located on the downstream of Bcl-2 family in the apoptosis pathway, Caspase-3 is the main executioner of apoptosis and is activated by upstream intrinsic or extrinsic pathways of apoptosis. The cleavage of Caspase-3 is a typical hallmark of apoptosis. Interestingly, we found that BPDE increased apoptosis of Swan 71 cell through the Bax/Bcl-2/Caspase-3 signaling pathway.

Pregnancy is a complex process comprising implantation, decidualization, placentation, and finally the process of parturition. The successful implantation of embryo is steered by the processes of apposition, adhesion, attachment, and penetration. During the implantation, blastocysts are attached to the subepithelial stroma, followed by penetration of trophoblast cells through the luminal epithelium and basal lamina into the stroma [35]. In this case, migration and invasion of trophoblasts play an important role in embryo implantation and hence the pathogenesis of miscarriages. Long noncoding RNA *PVT1* is decreased in villi tissue samples from spontaneous abortion, and *PVT1* regulates trophoblast migration [36]. EIF5A1 promotes trophoblasts migration and invasion via the integrin/ERK signaling pathway, and EIF5A1 downregulation plays a role in recurrent miscarriages [37]. Hence, we hypothesized that BaP and BPDE may induce miscarriage through the dysfunction of migration in trophoblasts. Human trophoblast migration and invasion are reported to be regulated by a series of signaling mediators, including FAK, G proteins, MAPKs, and PI3K/AKT [38]. Previous studies have reported that the PI3K/AKT signaling pathway regulates mobilization, migration, angiogenesis, vessel formation, metabolism, transcription, cell survival, growth, and tumorigenesis [39]. MMPs are a family of secreted or transmembrane gelatinase that could digest ECM [40]. Among the MMPs, MMP2 and MMP9 are highly expressed in trophoblasts and crucial for trophoblast invasion [41]. The XCL1-XCR1 chemokine pathway promotes trophoblast invasion by increasing MMP-2 and MMP9 activity in human first trimester placenta [42]. Our *in vitro* experiment results indicated that BPDE reduced migration of trophoblast cells in a dose-dependent manner through the PI3K/AKT/MMP2 signaling pathway. This is consistent with the observation from the studies of villous explants in woman with RPL and in BPDE exposure.

In conclusion, our study showed that exposure to BaP impaired the normal pregnancy in mice during early pregnancy. It is interesting to note that villous explants from the RPL group showed reduced migration and invasion. In case of the mechanisms involved in the effect of BaP on villi/EVT, migration and apoptosis of trophoblast and the signaling pathway involved in those cell functions were studied. The above findings provide the basis for our future work. Considering the toxic effect of BaP as demonstrated in our results, it is imperative to curtail BaP emissions in the environment and protect fertile women from environmental and occupational exposure.

## Figures and Tables

**Table 1:** List of PCR primers.

	Forward	Reverse
PIK3CA	5′-GAGTACCTTGTTCCAATCCCAG-3′	5′-TTCCTCTTTAGCACCCTTTCG-3′
AKT1	5′-TCTATGGCGCTGAGATTGTG-3′	5′-TCTTAATGTGCCCGTCCTTG-3′
MMP2	5′-GTCTTCCCCTTCACTTTCCTG-3′	5′-ACCACATCTTTCCGTCACTG-3′
GAPDH	5′-GGAGCGAGATCCCTCCAAAAT-3′	5′-GGCTGTTGTCATACTTCTCATGG-3′

**Table 2:** Day 8 BaP exposure of pregnant mice exhibited increased abortion in a dose-dependent manner.

Concentration of BaP (mg/kg)	Day of sacrifice	Abortion rate
0	8	20.0% (1/5)
0.2	8	66.7% (4/6)^∗^
2	8	100% (3/3)^∗∗^

**Table 3:** Day 10 BaP exposure of mice exhibited increased abortion and abnormal fetus percentage in a dose-dependent manner.

Concentration of BaP (mg/kg)	Day of sacrifice	Abortion rate	Percentage of abnormal fetuses
0	10	12.5% (1/8)	0
0.05	10	40.0% (2/5)	0
0.1	10	55.6 (5/9)	12.5%^∗^
0.2	10	75.0% (6/8)^∗^	26.7%^∗^

## Data Availability

The table and figure data used to support the findings of this study are included within the article.
